# Selective oxidation of B800 bacteriochlorophyll *a* in photosynthetic light-harvesting protein LH2

**DOI:** 10.1038/s41598-019-40082-y

**Published:** 2019-03-06

**Authors:** Yoshitaka Saga, Kiyoshiro Kawano, Yuji Otsuka, Michie Imanishi, Yukihiro Kimura, Sayaka Matsui, Hitoshi Asakawa

**Affiliations:** 1 Department of Chemistry, Faculty of Science and Engineering, Kindai University, Higashi-Osaka, Osaka, 577-8502 Japan; 2 Graduate School of Agricultural Science, Kobe University, Kobe, 657-8501 Japan; 3 Graduate School of Natural Science and Technology, Kanazawa University, Kanazawa, 920-1192 Japan; 4 Nanomaterials Research Institute, Kanazawa University, Kanazawa, 920-1192 Japan; 5 Nano Life Science Institute (WPI-NanoLSI), Kanazawa University, Kanazawa, 920-1192 Japan

## Abstract

Engineering chlorophyll (Chl) pigments that are bound to photosynthetic light-harvesting proteins is one promising strategy to regulate spectral coverage for photon capture and to improve the photosynthetic efficiency of these proteins. Conversion from the bacteriochlorophyll (BChl) skeleton (7,8,17,18-tetrahydroporphyrin) to the Chl skeleton (17,18-dihydroporphyrin) produces the most drastic change of the spectral range of absorption by light-harvesting proteins. We demonstrated *in situ* selective oxidation of B800 BChl *a* in light-harvesting protein LH2 from a purple bacterium *Rhodoblastus acidophilus* by 2,3-dichloro-5,6-dicyano-1,4-benzoquinone. The newly formed pigment, 3-acetyl Chl *a*, interacted with the LH2 polypeptides in the same manner as native B800. B850 BChl *a* was not oxidized in this reaction. CD spectroscopy indicated that the B850 orientation and the content of the α-helices were unchanged by the B800 oxidation. The nonameric circular arrangement of the oxidized LH2 protein was visualized by AFM; its diameter was almost the same as that of native LH2. The *in situ* oxidation of B800 BChl *a* in LH2 protein with no structural change will be useful not only for manipulation of the photofunctional properties of photosynthetic pigment-protein complexes but also for understanding the substitution of BChl to Chl pigments in the evolution from bacterial to oxygenic photosynthesis.

## Introduction

Light-harvesting antenna proteins play crucial roles in the primary process in photosynthesis. In these proteins, cyclic tetrapyrrole pigments such as chlorophyll (Chl) and bacteriochlorophyll (BChl) are embedded in the protein matrix and engaged in the efficient capture of photons and the transfer of excitation energy to the reaction center complexes. The spectral coverage of pigments in light-harvesting proteins is the basis for efficient photosynthetic ability in photosynthetic organisms. Generally, oxygenic photosynthetic organisms utilize Chl pigments (17,18-dihydroporphyrin), whereas purple photosynthetic bacteria and heliobacteria exploit BChls *a*, *b*, and *g* (7,8,17,18-tetrahydroporphyrin)^1–3^. The skeletons of (B)Chl pigments are primarily responsible for their spectral properties. The lowest energy absorption bands (Q_y_ bands) of the 17,18-dihydroporphyrin pigments are positioned at approximately 100-nm shorter wavelength regions than those of the 7,8,17,18-tetrahydroporphyrin pigments.

Light-harvesting complex 2 (LH2), which is a peripheral antenna protein in purple photosynthetic bacteria, has BChl *a* and carotenoids as light-harvesting pigments. LH2 proteins have symmetric circular arrangements of pigment-polypeptide subunits^4–6^. BChl *a* pigments in LH2 proteins are classified into two types termed as B800 and B850 due to their Q_y_ bands around 800 and 850 nm. B800 BChl *a* is present as the monomeric form in LH2 proteins. In contrast, B850 BChl *a* pigments form a dimer in one subunit composed of transmembranous α- and β-polypeptides and excitonically interact with each other in the circular arrangement. The excitation energy captured by B800 BChl *a* is transferred to B850 BChl *a* in LH2 proteins^7–9^.

Changing BChl *a* to Chl-type pigments in bacterial photosynthetic proteins is one excellent strategy to control light-harvesting and energy transfer abilities. This is helpful not only for the elucidation of the mechanisms of energy transfer and photoprotection in the target photosynthetic proteins but also for the development of novel photofunctional proteins. In this regard, the exchange of B800 BChl *a* with Chl pigments in LH2 proteins by B800-removal and the subsequent insertion of exogenous Chls have been studied^10–17^.

In contrast to the exchange of B800 BChl *a*, there is little information on *in situ* conversion of BChl *a* into Chl-type pigments in LH2 proteins. Especially, no information is available about the isolation and the detailed characterization of LH2 proteins whose B800 BChl *a* is selectively converted to Chl pigments by *in situ* oxidation, although BChl oxidation has been reported in studies on photoprotection in light-harvesting proteins in purple bacteria^18–24^ and the functional roles of BChl pigments in proteins in green sulfur bacteria^25^ and heliobacteria^26^. Generally bacteriochlorin pigments (7,8,17,18-tetrahydroporphyrins) are chemically oxidized by 2,3-dichloro-5,6-dicyano-1,4-benzoquinone (DDQ) to give corresponding chlorins (17,18-dihydroporphyrins)^27^. We apply this reaction to *in situ* conversion of B800 BChl *a* in LH2 as a novel methodology to regulate the photofunctional abilities.

**Figure 1:**
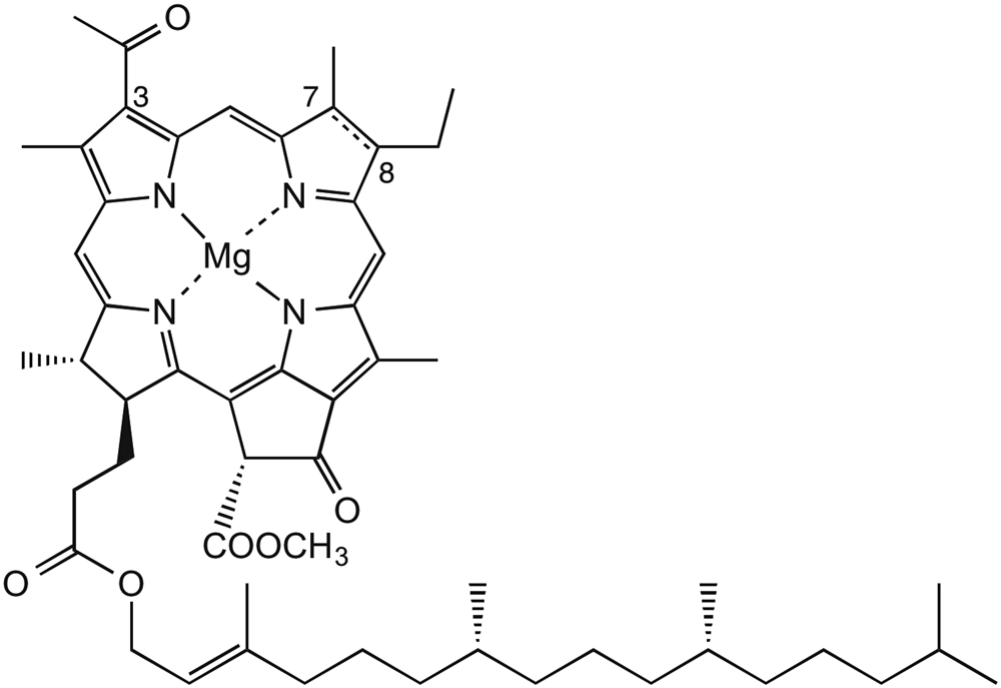
Molecular structures of BChl *a* (single bond between C7 and C8) and 3-acetyl Chl *a* (double bond between C7 and C8).

In this study, we performed selective conversion of B800 BChl *a* to a corresponding chlorin pigment called 3-acetyl Chl *a* (Fig. 1) in LH2 protein isolated from a purple photosynthetic bacterium *Rhodoblastus* (*Rbl*.) *acidophilus* by oxidation with DDQ. In contrast to the complete oxidation of B800 BChl *a*, no oxidation of B850 BChl *a* occurred. The newly formed pigment, 3-acetyl Chl *a*, was accommodated in the B800 site in essentially the same manner as native BChl *a* and transferred the excitation energy to B850 BChl *a*. Circular dichroism (CD) spectroscopy and atomic force microscopy (AFM) indicated that the protein structure was preserved even if B800 BChl *a* is converted to 3-acetyl Chl *a*.

## Results

### Spectral properties

**Figure 2:**
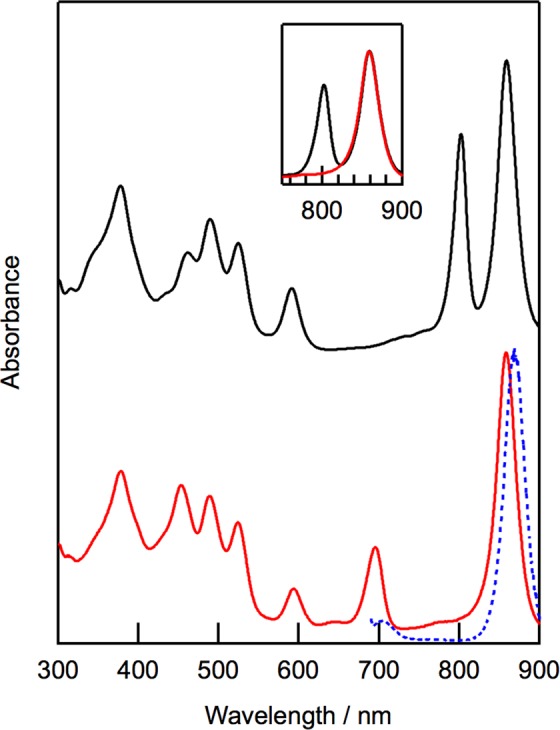
Electronic absorption spectra of native LH2 (black) and oxidized LH2 (red) in 20 mM Tris-HCl buffer containing 0.1% *n-*dodecyl-*β*-D-maltoside (pH = 8.0). Spectra were normalized at Q_y_ peaks of B850 BChl *a*. Fluorescence emission spectrum of oxidized LH2 by excitation at 680 nm (blue) is shown with its absorption spectrum. Insert shows overlapped absorption spectra of native LH2 and oxidized LH2 in Q_y_ region.

Incubation of native LH2 with DDQ followed by purification with anion exchange chromatography gave LH2 protein whose B800 BChl *a* was oxidized (denoted as oxidized LH2). We compared the electronic absorption spectrum of this protein with that of the native LH2 (Fig. 2). The native LH2 protein showed two intense Q_y_ absorption bands of B800 and B850 BChl *a* at 802 and 859 nm. In contrast, the oxidized LH2 protein had no Q_y_ band of B800 BChl *a* but exhibited a new absorption band at 694 nm. This peak position was the same as that of 3-acetyl Chl *a*, which was inserted into the B800 site in B800-depleted LH2^16^. The peak position and the bandwidth of the Q_y_ band of B850 BChl *a* in the oxidized LH2 was identical to those in the native LH2 (Fig. 2, insert). The Soret band of 3-acetyl Chl *a* in the oxidized LH2 overlapped with the absorption bands of a carotenoid (rhodopin glucoside) at 453 nm.

The Q_x_ band of BChl *a* in the oxidized LH2 protein was detected at 593 nm, which was slightly red-shifted from the Q_x_ band in the native LH2 at 591 nm. This shift is ascribable to the disappearance of the Q_x_ band of B800 BChl *a* by the conversion of this pigment to 3-acetyl Chl *a*, since the Q_x_ band of BChl *a* is derived from both B800 and B850 BChl *a*, whose Q_x_ positions slightly differed, in native LH2^16^.

The absorption bands of rhodopin glucoside in the native LH2 were positioned at 524, 489, and 461 nm. The positions of the two former bands in the oxidized LH2 were the same as those in the native LH2. Note that the band of rhodopin glucoside at 461 nm overlapped with the Soret band of 3-acetyl Chl *a* in the spectrum of the oxidized LH2. The coincidence of the peak positions of rhodopin glucoside between the oxidized LH2 and the native LH2 indicates that 3-acetyl Chl *a* is properly accommodated in the B800 site, since its peak positions are sensitive to the presence of the chlorophyllous pigment in the B800 site in LH2^10,11^.

**Figure 3:**
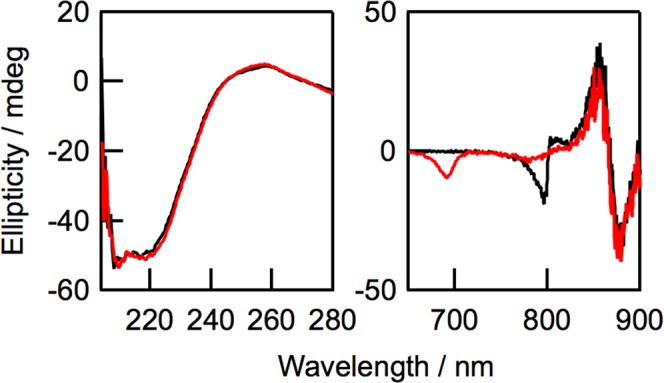
CD spectra of native LH2 (black) and oxidized LH2 (red) in UV (left) and Q_y_ regions (right) in 20 mM Tris-HCl buffer containing 0.1% *n-*dodecyl-*β*-D-maltoside (pH = 8.0). Q_y_ absorbance values of B850 BChl *a* in LH2 samples used for measurements were 0.9.

A CD spectrum of the oxidized LH2 is compared with that of the native LH2 (Fig. 3). The negative signal of B800 BChl *a* in the native LH2 around 800 nm disappeared accompanying a new negative signal around 695 nm in the CD spectrum of the oxidized LH2 (Fig. 3, right). The position of this new CD signal corresponds to the Q_y_ absorption band of 3-acetyl Chl *a*. The reversed *S*-shaped CD signal of B850 BChl *a* in the CD spectrum of the oxidized LH2 between 820 and 900 nm closely resembled that of the native LH2, indicating that the treatment of the native LH2 with DDQ barely disturbed the orientation and electronic structures of B850 BChl *a*. The shape and intensity of the negative CD signal in the oxidized LH2 in the UV region is also quite analogous to that of the native LH2, indicating that the content of the α-helices in the oxidized LH2 was identical to that in the native LH2. Therefore, the local structure around B850 BChl *a* and the secondary structure in LH2 were preserved even if B800 BChl *a* was oxidized *in situ*.

Fluorescence spectroscopy demonstrates the intracomplex energy transfer from the oxidized pigments in the B800 sites to B850 BChl *a*. The predominant excitation of 3-acetyl Chl *a* in the oxidized LH2 produced a strong emission from B850 BChl *a* at 868 nm with little emission from 3-acetyl Chl *a* around 700 nm (Fig. 2, blue curve). The apparent fluorescence quantum yields of B850 BChl *a* by the excitation of 3-acetyl Chl *a* in the oxidized LH2 was 8.7 ± 0.6% (the average and standard deviation of five samples). This value is consistent with that of native LH2 by the excitation of B800 BChl *a* (9.1%)^17^. These results indicate that 3-acetyl Chl *a* in the B800 sites can efficiently transfer the excitation energy to B850 BChl *a* in the oxidized LH2 like in the native LH2.

### Pigment analysis

**Figure 4:**
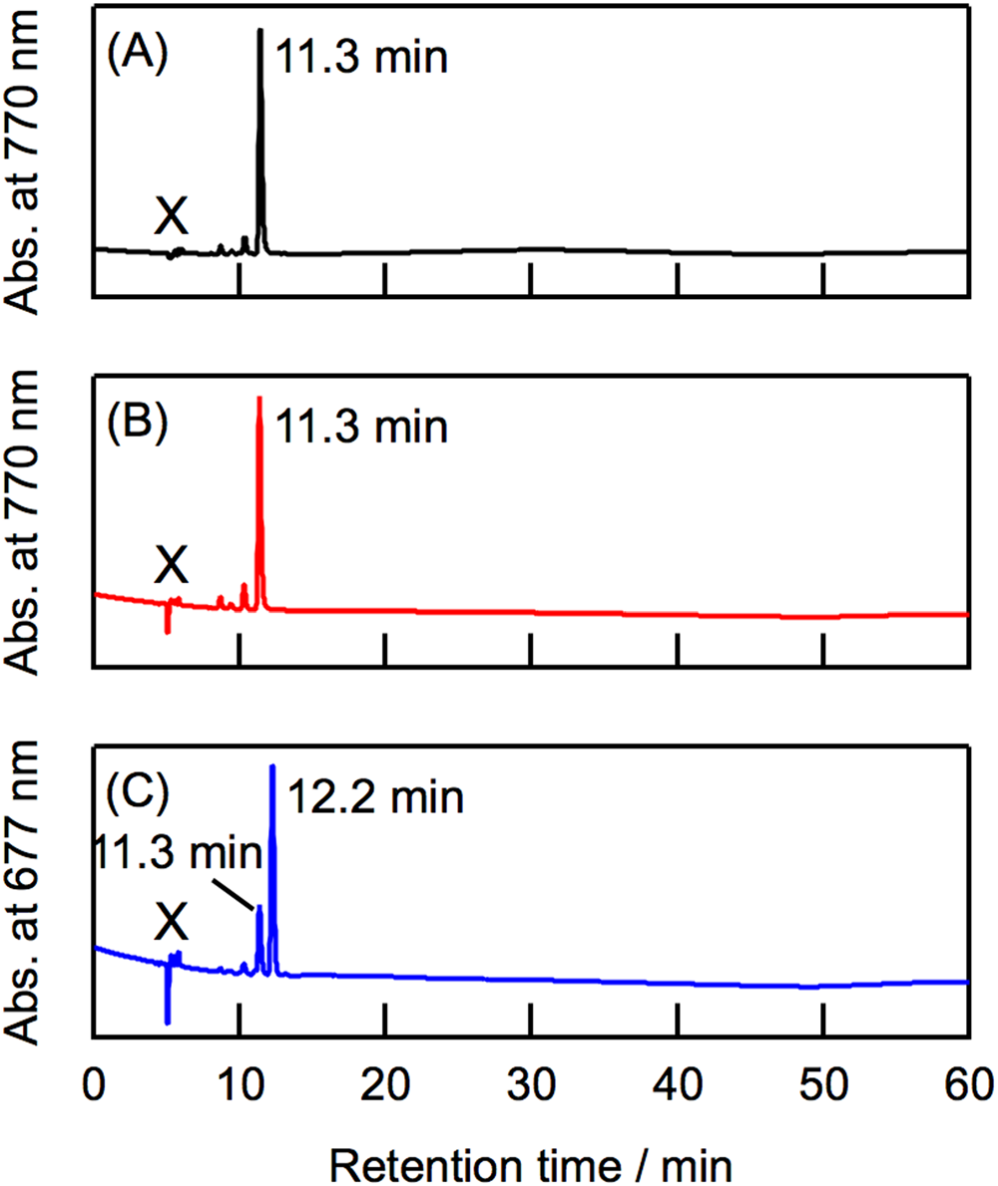
HPLC chromatograms of chlorophyllous pigments in native LH2 (**A**) and oxidized LH2 (**B**,**C**). Chromatograms (**A**–**C**) were recorded at 770, 770, and 677 nm, respectively. LH2 proteins were directly injected and eluted on a reverse-phase column 5C_18_-AR-II (6 mm i.d. × 250 mm) with a guard column 5C_18_-AR-II (4.6 mm i.d. × 10 mm) with methanol at the flow rate of 1.0 mL min^–1^. Signals denoted by × were due to direct injection of LH2 solutions.

Chlorophyllous pigments in the oxidized LH2 protein were analyzed by reverse-phase HPLC. In the chromatogram of the pigments in the oxidized LH2, which were detected at 770 nm, a major fraction was eluted at 11.3 min (Fig. 4B). The retention time of this fraction was identical to that of BChl *a* in the native LH2 (Fig. 4A). Electrospray ionization mass spectrometry (ESI-MS) also confirmed that this pigment was BChl *a*; the observed ion peaks (*m*/*z* = 911.6 and 933.6) were identical to the calculated values of BChl *a* for [MH^+^] and [M + Na^+^] (911.55 and 933.54). A new fraction was observed at 12.2 min in the 677-nm detected chromatogram of the chlorophyllous pigments in the oxidized LH2 (Fig. 4C). This fraction exhibited Soret and Q_y_ bands at 441 and 685 nm, respectively, in its on-line absorption spectrum. The ion peaks of this fraction by ESI-MS were detected at *m*/*z* = 909.6 and 931.6, which correspond to the calculated values of 3-acetyl Chl *a* for [MH^+^] and [M + Na^+^] (909.54 and 931.52). Few fractions, which are ascribable to the by-products *via* oxidation, were observed in Fig. 4B,C. Note that further oxidized porphyrin-type pigment of BChl *a*, namely 3-acetyl protochlorophyll *a*, is absent in the current HPLC analysis. These results indicate that 3-acetyl Chl *a* is predominantly formed by the *in situ* oxidation of the native LH2 protein.

### Resonance Raman spectra

**Figure 5:**
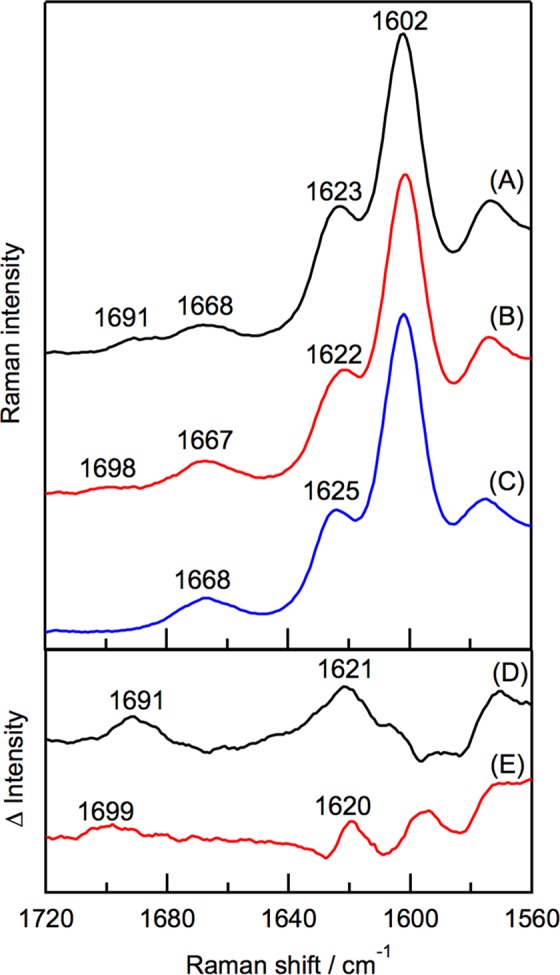
Resonance Raman spectra of native LH2 (**A**), oxidized LH2 (**B**) and B800-depleted LH2. (**C**) Difference spectra (**D**,**E**) were obtained by subtracting spectrum C from spectra A and B, respectively.

The interactions of 3-acetyl Chl *a* with the polypeptides in the oxidized LH2 protein were examined by resonance Raman spectroscopy. The native LH2 showed the 3-C=O stretching vibrational bands of BChl *a* at 1623 cm^−1^ (Fig. 5A). The 3-C=O band of B800 BChl *a* overlapped with that of B850 BChl *a*; the difference Raman spectrum between the native LH2 and the B800-depleted LH2 revealed the 3-C=O band of B800 BChl *a* at 1621 cm^−1^ (Fig. 5D). This position indicates that the 3-acetyl group in B800 BChl *a* is hydrogen-bonded with the β-Arg20 in LH2^12^. The oxidized LH2 protein exhibited the 3-C=O stretching vibrational band of 3-acetyl Chl *a* at 1620 cm^−1^ in the difference Raman spectrum between the oxidized LH2 and the B800-depleted LH2 (Fig. 5E), indicating that the 3-acetyl group in the oxidized pigment is also hydrogen-bonded with the LH2 polypeptide.

The position of the Raman signal of the 13-C=O stretching vibrational band of 3-acetyl Chl *a* in the oxidized LH2 at 1698 cm^−1^ was analogous to that of B800 BChl *a* in the native LH2 (Fig. 5A,B), indicating that the 13-keto group in 3-acetyl Chl *a* is free from hydrogen-bonding with polypeptides like native BChl *a* in the B800 site^12^. Therefore, 3-acety Chl *a* is accommodated in the B800 site in essentially the same manner as native BChl *a* even if B800 BChl *a* was oxidized.

The signal in the Raman spectrum of the oxidized LH2 protein at 1667 cm^−1^ is assigned to the 13-C=O stretching vibrational band of B850 BChl *a* (Fig. 5B). This position is almost the same as those in the native LH2 and the B800-depleted LH2 (Fig. 5A,C). The signals at 1602 cm^−1^ are assigned to the CC stretching modes of the methine bridges of BChl *a* and 3-acetyl Chl *a*.

### AFM observations

**Figure 6:**
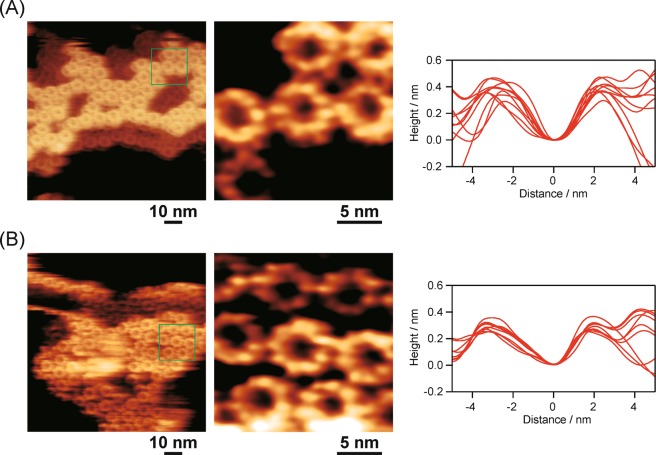
FM-AFM images of native LH2 (**A**) and oxidized LH2 (**B**) adsorbed on mica taken in 20 mM Tris buffer containing 150 mM NaCl (pH 8.0). Left: wide images. Middle: locally enlarged images of single LH2 proteins. Right: overlapped height-profiles of ten proteins.

The overall structure of the oxidized LH2 protein was clearly visualized with a home-built frequency modulation AFM (FM-AFM) in a buffer solution. The ring structure, which is comprised of nine subunits, in the oxidized LH2 was quite analogous to that of the native LH2 (Fig. 6, left and center), indicating that no deformation of the LH2 ring structure was induced by the oxidation of B800 BChl *a* in the protein. The averaged top-to-top distances of the LH2 ring in both the native and oxidized LH2 were estimated to be 5.2 ± 0.3 and 5.1 ± 0.2 nm (the averages and standard deviations of ten samples) by the height-profiles obtained from the AFM images (Fig. 6, right). Therefore, the ring diameter in the LH2 protein was unchanged by the *in situ* conversion of BChl *a* to 3-acetyl Chl *a* in the B800 sites.

## Discussion

In this study, we first demonstrated the selective conversion of B800 BChl *a* to 3-acetyl Chl *a* in LH2 protein by *in situ* oxidation with DDQ and characterized the oxidized protein by spectroscopic and AFM measurements. B800 BChl *a* was completely changed to 3-acetyl Chl *a* without oxidation of B850 BChl *a*. 3-acetyl Chl *a* was accommodated in the B800 site in essentially the same manner as B800 BChl *a*. The secondary structure and the nonameric circular arrangement of the LH2 protein remained *via* this oxidation.

Although chemical, photochemical, and electrochemical oxidation of isolated light-harvesting proteins and chromatophores in some purple photosynthetic bacteria has been reported^18–24^, a mixture of oxidized products was formed from BChl *a* in the light-harvesting proteins and site-specific oxidation of BChl *a* in LH2 was scarcely observed. These results are in sharp contrast to our results, which clearly indicate that only B800 BChl *a* was exclusively converted to 3-acetyl Chl *a*. The differences are mainly ascribable to the oxidants used for the chemical oxidation of LH2 proteins; ferricyanide was commonly used in previous studies^18,21–23,25^. Ferricyanide produces a cation radical of BChl *a*, which is subsequently converted to oxidized products, since ferricyanide acts as a one-electron oxidant. Such reactions *via* the cation radical reduce both the selectivity of the oxidized products and the B800/B850 oxidation inside LH2. On the contrary, DDQ used in the current study can withdraw two hydrogen atoms from the bacteriochlorin and chlorin macrocycles^27–29^. This feature is appropriate for the product selectivity by oxidation of BChl *a* in LH2 protein. In addition, DDQ does not react with B850 BChl *a* embedded in the protein matrix and only oxidizes B800 BChl *a* that is exposed on the outside of the protein scaffold, producing the site-selectivity for oxidation of B800 BChl *a* in LH2. Preferential photooxidation of B850 BChl *a* in LH2 from a purple sulfur bacterium *Ectothiorhodospira haloalkaliphila* was reported very recently^24^. This B850 photooxidation induced the blue-shift of the Q_y_ band of B850 BChl *a*, and the blue-shift was reproduced by simulations of electronic absorption spectra of successive numbers of oxidized pigments in the B850 sites in LH2. In contrast, the current chemical oxidation produces no shift of the B850 Q_y_ band (Fig. 2), supporting no oxidation of B850 BChl *a* in LH2.

The current study will shed new light on the substitution from BChl (7,8,17,18-tetrahydroporphyrin) to Chl pigments (17,18-dihydroporphyrin) in the evolution of photosynthetic organisms. Phylogenic analyses of the genes of the enzymes for (B)Chl synthesis suggest that ancient photosynthetic organisms might possess BChl pigments, although the first appearance of chlorophyllous pigment (Chl or BChl) in the evolutional history has not been accurately unraveled^30–36^. Oxygenic conditions might be one type of evolutionary pressure that caused the shift from BChl to Chl pigments by changing from anoxygenic to oxygenic organisms. Indeed, our study demonstrates that an oxidant can change BChl *a* bound to LH2 protein to a corresponding Chl pigment and the formed Chl pigment can engage in light-harvesting. These results are in line with a working hypothesis called the acquisition and discrimination model for pigment-binding proteins proposed by Mimoro and Tanaka^37^, in which the incorporation of a new pigment into pre-existing proteins leads to the acquisition of new genes for the biosynthesis of the new pigment and the subsequent evolution of a protein moiety. In the case of evolution from BChl *a* to Chl *a*, the loss of the genes of the enzymes for the reduction of the chlorin macrocycle and the conversion of the 3-substituent from the vinyl to acetyl groups might be promoted by the oxidation of BChl *a* that is bound to ancestor photosynthetic proteins.

In conclusion, B800 BChl *a* can be selectively converted to a corresponding chlorin-type pigment, 3-acetyl Chl *a*, in LH2 protein from *Rbl*. *acidophilus* without perturbation to the protein structure by oxidation with DDQ. This conversion allows the LH2 protein to collect red-light, which is not absorbed by native LH2 even though the photon number in the red region is maximum in all the wavelength region of the photons that reached the surface on the earth. The *in situ* oxidation of BChl *a* in LH2 protein will be useful for engineering photofunctions in natural light-harvesting proteins and for understanding the alteration from BChl pigments in anoxygenic photosynthetic bacteria to Chl pigments in oxygenic organisms in the evolution of photosynthesis.

## Materials and Methods

### Materials

Native LH2 protein was isolated from the cultured cells of a purple photosynthetic bacterium *Rbl*. *acidophilus* DSM145 according to previous reports^38,39^. B800-depleted LH2 protein was prepared as reported elsewhere^39^. DDQ and acetone were purchased from Wako Pure Chemical Industries, Ltd. (Osaka, Japan) and used without further purification. DDM was purchased from Dojindo Laboratories, Co. (Kumamoto, Japan).

### Preparation of oxidized LH2

A solution of native LH2 in 20 mM Tris buffer containing 0.1% DDM (pH 8.0) was adequately desalted and mixed with 1/10 volume of an acetone solution of DDQ. The relative molar ratio of DDQ to native LH2 was 2000 in the mixed solution. The solution was incubated at 35 °C in the dark. After disappearance of the B800 Q_y_ band using electronic absorption spectral analysis, DDQ was roughly removed by ultrafiltration using Amicon centricon concentrators (30 kDa cutoff, Merk Millipore Ltd., Cork, Ireland). Oxidized LH2 was purified by anion exchange column chromatography using Whatman DE52 resin (GE Healthcare, Little Chalfont, U.K.), followed by ultrafiltration using Amicon centricon concentrators (30 kDa cutoff, Merk Millipore Ltd., Cork, Ireland).

### Spectroscopic measurements

Electronic absorption and CD spectra of LH2 proteins were measured with a Shimadzu UV-2450 spectrophotometer (Shimadzu, Kyoto, Japan) and a JASCO J-820 spectropolarimeter (JASCO, Tokyo, Japan). Fluorescence emission spectra were measured with a Hitachi F-7100 spectrophotometer (Hitachi, Tokyo, Japan). The apparent fluorescence quantum yields of B850 BChl *a* were measured by a Hamamatsu C9920-03G fluorescence measurement system (Hamamatsu Photonics, Shizuoka, Japan) from emission between 840 and 950 nm.

### HPLC analysis of chlorophyllous pigments

LH2 proteins in 20 mM Tris buffer containing 0.1% DDM (pH = 8.0) were concentrated by ultrafiltration using Amicon centricon concentrators (30 kDa cutoff, Merk Millipore Ltd., Cork, Ireland), followed by the injection of the concentrated LH2 solution into a HPLC system comprised of a Shimadzu LC-20AT pump, a SPD-M20A photodiode array detector, and a Shimadzu CBM-20A system controller (Shimadzu, Kyoto, Japan). ESI-MS analysis was performed with a Shimadzu LCMS-2020 system (Shimadzu, Kyoto, Japan).

### Resonance Raman spectroscopy

An aliquot of a LH2 solution was deposited on a stainless plate and dried with nitrogen. Excitation beam (355 nm) from a Nd:YAG laser was focused onto the sample film through ×40 objective lens. The laser intensity at the sample surface was adjusted to ∼0.4 mW and backscattering from the sample was collected at 25 °C. Each spectrum was accumulated for 30 s at a single spot to alleviate sample degradation. To improve the S/N ratio, 25–80 spectra at different points were averaged.

### AFM measurements

A 100 μL of a solubilized LH2 solution in 20 mM Tris buffer containing 0.1% DDM and 150 mM NaCl (pH 8.0) was deposited to a cleaved mica substrate with a diameter of 12 mm (SPI Supplies, West Chester, PA, USA), followed by standing for 30 min at room temperature in the dark. Then the sample was rigorously rinsed with 20 mM Tris buffer containing 150 mM NaCl (pH 8.0) and observed in 20 mM Tris buffer containing 150 mM NaCl (pH 8.0) by a home-built FM-AFM^40–42^ with a silicone cantilever (PPP-NCHAuD, Nanoworld, Headquarters, Switzerland), which had a nominal spring constant of 42 N/m. The typical resonance frequency and the Q factor in an aqueous buffer solution were 150 kHz and 7. A commercially available AFM controller (ARC2, Asylum Research, Santa Barbara, CA, USA) was used to control the FM-AFM system.

## Data Availability

Data generated or analyzed during the current study are included in this published article and are available from the corresponding author on reasonable request.
